# Community acceptance of COVID-19 and demystifying stigma in a severely affected population in Ghana

**DOI:** 10.4314/gmj.v55i2s.2

**Published:** 2021-06

**Authors:** Delia A Bandoh, Abraham Baidoo, Charles L Noora, Sally Quartey, Joseph A Frimpong, Ernest Kenu

**Affiliations:** 1 G.F.E.L.T.P, Department of Epidemiology and Disease Control, School of Public Health, University of Ghana, Legon, Accra, Ghana; 2 Tema Metro Health Directorate, Ghana Health Service, Tema, Ghana

**Keywords:** Stakeholders, COVID-19, Ghana, stigma, community

## Abstract

**Objective:**

We assessed the level of community acceptance of COVID-19, identified and implemented strategies to demystifying stigma in a severely affected population in Tema.

**Design and Setting:**

We conducted a cross-sectional study to assess stigma among the Tema community, then identified and implemented interventions to demystify COVID-19 stigma. We interviewed positive cases, their contacts, contact tracers, case management team members, and community members who shared their first hand experiences and knowledge on the current pandemic.

**Intervention:**

Based on the information received, we came up with ways of reducing stigma and implemented them in their community.

**Main Outcome:**

Stigma demystified

**Results:**

Cases and contacts reported being avoided, discriminated against, insulted or had derogatory words used on them by family, friends, work colleagues or the community. Cases and their contacts stated that stigmatisation was fueled by the presence of COVID -19 branded vehicles and security officials at their homes or workplaces. Stakeholder engagement, education and extensive sensitisation of community members were implemented to reduce stigma.

**Conclusion:**

We observed deeply entrenched stigma to COVID - 19 positive patients and their contacts in the community. Health care response mechanisms such as the presence of security personnel with contact tracers and case managers and the use of COVID -19 branded vehicles fueled stigma. A multifaceted approach through the engagement of key stakeholders, training of health workers and extensive education and community sensitisation was essential in reducing stigma.

**Funding:**

The COVID-19 outbreak response and writing workshop by the Ghana Field Epidemiology and Laboratory Training Programme (G.F.E.L.T.P.) was supported with funding from President Malaria Initiative – C.D.C., and Korea International Cooperation Agency (on C.D.C. CoAg 6NU2GGH001876) through AFENET

## Introduction

Coronavirus disease (COVID-19) is an infectious disease caused by a newly discovered coronavirus.^1^ A report made on 31st December 2019 to the World Health Organization (WHO)of acute respiratory illness in a cluster of people in Wuhan, China, was later confirmed to be due to the novel coronavirus (SARS-Cov-2). Subsequently, the WHO declared a pandemic due to the virus on 11th March 2020.

According to the WHO, the global case count of COVID-19 as of 1st July 2020 stood at over 10 million cases, with more than 500 thousand deaths. The Afri-can region contributes about 300 thousand of the global case counts, with over 600 thousand deaths.

In Ghana, the current case count as of 1st July 2020 was estimated as 18134, with 117 deaths, according to the Ghana Health Service.^2^

As there is still no cure for this disease, segregation from infected persons appears to be the only preventative solution. This has quite different impacts and consequences for local people.^2^ Though a novel disease across the globe, stigmatisation has become one of its issues. Scambler^3^ defined stigmatisation in relation to a discrediting remark that prevents the formation of healthy relationships and leads to social discrimination and alienation.

Many studies have suggested that stigma leads to social discrimination, linked to negative stereotypes and maybe a visible physical flaw or mark, a particular feature, situation or past event. Stigma is a combination of ignorance, prejudice and discrimination. Stigma is a word that has evaded clear, operational definition.^4^–^8^ It can be considered as a combination of three related problems: a lack of knowledge (ignorance and misinformation), negative attitudes (prejudice), and excluding or avoiding behaviours (discrimination).^9^–^14^ The combination of these three elements has a powerful force for social exclusion.^14^ Stigmatization continues to be associated with grief difficulties, depression, and suicidal thinking.^15^ Victims of various diseases (including bipolar disorder, HIV/ AIDS, Epilepsy, Ebola, Psoriasis) have been stigmatised by being disgraced or labelled as inferior over the years leading to devastating consequences. Stigma tends to breed suicidal ideation, depression, hopelessness, anxiety, unnecessary fear and social withdrawal.^16^

Since the outbreak of the novel disease, victims have been stigmatised across the world. These negative emotional consequences associated with these diseases have significantly affected the fight against reducing the burden of these diseases.^17^,^18^ There was a global opportunity to reduce the burden of stigmatisation associated with the disease right from its onset. However, the novelty of the disease concerning the clinical features and case fatality contributed to the stigmatisation of people who had the disease. This is mainly due to ignorance or misinformation about the disease. Initial reports from Wuhan and Europe where the pandemic was fast spreading suggested classical signs and symptoms of COVID-19 leading to high mortality. This created a sense of fear and anxiety across the world.

In Ghana, COVID-19 was believed by many to be a disease of the elite. Initial reports by contact tracing teams and case managers show that people were hesitant to relate to them due to fear and negative attitude and avoiding behaviour from community members after interaction with them. This is because there is a general notion that being isolated or under quarantine in our cultural context is strange and usually associated with witchcraft or punishment by the gods and thus leads to stigmatisation.^19^ The situation is even worse with branded COVID-19 vehicles and security personnel in households of victims.

Daily situational reports by the Ghana Health Service Contact Tracing Team indicates that the protective gear worn by health officials in attending to cases or suspects also breads serious anxiety for both victims and the community leading to avoiding behaviour from community members after the visit.

Pandemics like COVID affect physical health and have psychological implications at the individual, community, and country levels. This effect is worsened by the negative attitudes fueled by ignorance and fear, leading to stigmatisation. Reducing the effect of a stigma requires understanding the causes from the community's perspective. This paper documents the stigma related to COVID-19 in a severely affected population in the country and steps implemented to demystifying stigma.

## Methods

### Study design

We assessed COVID-19 related stigma among the Tema community and implemented interventions to demystify it. Four domains of stigma were assessed. This included; the feeling of rejection by family members and friends, the fear of death from the disease; the feeling of shame; and frustration. We interviewed positive cases, their contacts, contact tracers, case management team members, and community members who shared their first hand experiences and knowledge on the current pandemic. Based on the information received, we came up with ways of reducing stigma and implemented them in their community.

### Study Area

This study was conducted in the Tema Metropolitan Assembly located in the Greater Accra Region, which also houses Ghana's biggest seaport, the Tema Harbour. Tema is the country's largest industrial area with an estimated population of about 220,000. Due to the port and industrial activities, Tema serves as the most significant area of revenue generation for the country. As such, Government considers several discussions on economic matters before taking any action related to COVID-19. Thus, the ports continued to work during the partial lockdown of parts of the country, including Tema. At the time of the study, the total confirmed cases in the Tema metropolis was 1,500 persons with 2,936 suspected persons.

### Participants

All residents of Tema aged 18 years and above, regardless of sex who were stakeholders in Ghana's COVID-19 response, were eligible to participate once they gave their consent. They include cases, recovered cases, contacts, community members, opinion leaders, contact tracers, health workers and many more. Forty-six (46) participants were purposively selected and interviewed. They were made of 20 cases and ten contacts, and 16 community members.

### Assessing Stigma in the Community

All interviews were conducted as conversations were where respondents were allowed to share their experiences on stigma.

In-depth interviews were conducted for contacts. These interviews were conducted privately in the home of the contacts in places provided by the contact. Interviews were conducted by contact tracers trained to conduct in-depth interviews. Both interviewer and contacts were in face masks during the interview and ensured they were at least 2 meters apart. In-depth interviews for cases were conducted at the treatment centre. The interviews were conducted by case managers trained to conduct interviews. These interviews were privately conducted in a room with only the case and case manager. The case managers were appropriately dressed in personal protective clothing, and the cases were in a face mask. A distance of at least two meters was kept during interviews.

Focus group discussions were conducted at the municipal health directorate. Community members who agreed to participate were invited to the municipal health directorate. A maximum of eight community members was involved in the focus group discussion. The discussion was facilitated by the resident field epidemiologist and the health promotion officer. All were in face masks, and the sitting arrangement was at least one meter apart.

All interviews were done with COVID-19 prevention protocols being observed. The interaction covered action and behaviours perceived as stigma, self-stigma, family, friends, community and workplace stigmatisation. These were related to their ability to freely relate, particularly their health, social and financial needs. We examined various activities conducted by each team based on initial complaints by cases and contacts in the following areas: the use of ambulances to pick up cases and contacts at home and the feedback from the community, the inclusion of security officers in contact tracing and case management teams, disclosure of organisations where the cases were coming from, and the use of COVID-19 branded vehicles for home visits to cases and contacts. Major points from all interviews were jotted down and used during the analysis. The scope of the interaction was obtained from the review of daily contact tracing reports provided by the lead supervisor for the Tema Metropolis.

### Intervention to reduce stigma

After interactions with the various stakeholders, we identified major themes that came up in the conversations and solutions that had been suggested. The investigation team came up with some innovations to address issues of stigmatisation encountered by cases and contacts in the community based on the outcome of the interaction.

### Ethical considerations

We obtained ethical clearance from the Ghana Health Service Ethics review committee (GHS-ERC 006/05/20). Participants were assured that their experiences with COVID-19 cases and contacts and the feedback they got from them would be kept confidential. Physical distance was maintained throughout the face-to-face interviews while wearing nose masks and exercising all recommended COVID-19 national protocols. Interviews were conducted on phone in cases where in-person interviews were not possible.

## Results

### Reports on Stigma

Forty-six (46) participants were interviewed, aged between 20 and 49 years. Of this, 35 (77%) were females, 27 (58.7%) were married, 7 (15.2%) divorced and 12(26.1%) single.

### Confirmed cases

Cases reported that community members tagged them as positive cases and avoided them, refusing to sell to or buy from them even after recovery. They also called them names and made derogatory statements about them. Cases indicated that their superiors at the workplace expressed dissatisfaction with the way they were picked up to the isolation centres by the ambulance service. This created a scene, making community members easily identify their families (some unaware) with the infection. This, they felt, breached their confidentiality and exposed them to negative attitudes such as rejecting them and discrediting them. Some cases reported that community members threatened them not to come back home from the isolation center after recovery since they had brought COVID-19 to the community.

Some positive cases reported self-stigma. They had the guilt of having unknowingly transferred the infection to others because they had initially been contacts to cases but had not been informed or listed as contacts to these cases. These contacts were tested as part of enhanced screenings and got to know their exposure after testing positive. They felt guilty not having quarantined and exposed family members, colleagues, drivers, and other infection-related relations.

### Identified case contacts

Contacts to positive cases recounted the stigma arising after contact tracers visiting them for follow-up and testing in the presence of security personnel and with COVID-19 branded vehicles. After the visits, community members tagged them as people spreading COVID-19, refused anything to do with them, insulted them, shunned them, and some went to the extent of refusing to sell to them.

A family of five contacts to a positive case who was on admission at the treatment centre requested for contact tracing to be done without branded vehicles but to be followed-up mostly on the phone (as they had their thermometers to do their daily temperature checks) and no security personnel to accompany the contact tracing team to the neighbourhood. This was due to fear that they would be ostracised by the community members and called names. Some contacts refused to quarantine and refused contact tracers to follow them up for fear that neighbours, community members and colleague workers would get to know their status and not allow them to use the same facilities or discriminate against them. Contacts felt that being singled out in a compound house where they live communally and shared amenities caused the other house members to see them as a threat to their lives and livelihoods. Due to fear of experiencing negative attitudes and insecurity, some refused to give full personal details so that the contact tracers would not locate their houses. Contacts of cases who had tested positive in some factories requested to be quarantined away from home for fear of being treated badly in their community.

### The behaviour of cases towards the case management team

The case management team received reports from positive cases who did not want to be moved to isolation centres though they could not self-isolate due to stigma. There were reports of cases who refused subsequent engagements, picked up calls and relocated from their places of residence to avoid stigmatisation. Some cases were in denial of their positive status on account of this.

### The behaviour of contacts towards contact tracing teams

Contact tracers encountered hostility from contacts of positive cases. They received verbal threats and abuse from contacts who either demanded to know the cases they had come into contact with or not to associate themselves with the contact tracing activity due to fear of stigma. An Assemblyman recounted having been threatened by members of his community after he led contact tracers to his community to screen contacts to positive cases.

The households screened were avoided, called names and discriminated against by the community, and some refused to retest after their quarantine period. The community pushed the Assemblyman to reveal the identity/ identities of the case(s) for which they were being followed up and tested. Contact tracers were attacked in the community and prevented from carrying out the retesting exercise. The Assemblyman refused to accompany contact tracers in a subsequent visit for fear of attack.

### Key Measures Implemented to Demystify Stigma in the Community

#### Education of key community stakeholders

As part of the community engagement, key stakeholders, including security officers from the Ghana Immigration Service, Ghana Police Service, the Military, Health officials from the Tema municipal health directorate, Traditional leaders, heads of organisations, Tema Municipal Assembly, Municipal Coordinating Director and Assemblymen were contacted and a meeting held with them. The Investigation team also engaged market leaders and transport union leaders of Tema. These meetings were held to educate these community leaders on COVID-19 prevention protocols, signs and symptoms, issues of stigma and its effect on the community. During these meetings, we discussed issues of fear about COVID-19, everyone being at risk of being infected and how to provide social support for recovered cases when they return to the community.

#### Training of health workers involved in the response

Counsellors, including psychiatric nurses, were trained on how to visit positive cases at home and disclose results to them, follow up positive cases with the contact tracers (in teams), to list contacts for follow up and testing. The counsellors assisted in counselling of identified contacts who posed a challenge to contact tracers. Health promotion officers in the Metro Health Directorate were also trained to carry out community education and sensitisation on COVID-19 and stigma related issues.

Contact tracers were also trained to respect confidentially of cases and contacts and how to engage entire households on a compound when visiting contacts to avoid community members from tagging particular people. They were also trained to engage community members who approach them and answer COVID-19 related questions they raised. The resident field epidemiologist and psychologist facilitated four training sessions and was supported by the disease control officer and health information officer. All facilitators assisted in the training of all the health teams involved in the response.

#### Community education and sensitization

We embarked on educating and sensitising key stakeholders and victims of COVID-19 to help reduce stigma in the community. Educational messages on awareness were drafted with the support of the G.F.E.L.T.P. and played throughout the community utilising the van. This helped in demystifying stigma issues. Some of the key messages include “we are all at risk, don't be judgmental”, the need to mind the kind of language used on cases and contacts, preventive measures for COVID-19. During these sessions, people were allowed to ask questions that bothered them, and they were addressed to reduce misinformation and ignorance.

#### Reorganising case management and contact tracing methods

Case management teams were formed to manage positive cases of self-isolation at home. This was composed of a Medical officer, nurses, counsellors, psychiatric nurses and laboratory personnel. Screening sites were set up for all retest cases and contacts who preferred screening outside their homes. These were dubbed “meet me there spots”. Positive cases were also picked up at agreed designated points outside their communities. Branded vehicles were used with discretion, and there was a limitation of the use of the security personnel, especially the immigration in contact tracing activities.

Counselling sessions were organised at the screening sites for suspected cases before laboratory samples for testing were taken. Once test results were ready, another session of counselling was conducted to declare results to them. Thus COVID-19 pre-test and post-test counselling were instituted at the screening sites.

## Discussion

There is still a lack of information on COVID-19 due to its novel nature. Therefore, it has created fear and panic in individuals, communities and nations globally following its socio-economic impact on individuals and society. This is further challenged by the psychosocial implications of stigmatisation of cases and contacts and healthcare staff. We assessed stigma in a community severely affected by COVID-19 and came up with measures to demystify stigma.

Our study found that COVID-19 cases felt stigmatised by their family and community members by the negative treatment they received. This was even worse after they had been declared recovered and returned home from isolation centres. Similar situations have been recorded in Nigeria where communities and even family members stigmatised recovered individuals.^20^

For fear of being rejected, derogatory words used on them and family members being physically and emotionally attacked, some positives refused isolation. This implies that out of fear of being stigmatised, cases would continue to remain in the community and unconsciously spread the virus to others instead of seeking treatment. Stigmatising cases affects the individuals and affects the entire response to fighting against the pandemic. Stigma can therefore lead to delay in seeking health care and prevent people from practising healthy behaviours.^21^,^22^ Given the trend of COVID-19 and its current spread, late reporting due to fear and refusal to be isolated or conform to the standard protocol can lead to the entire response system breaking down and an upsurge in the cases.

We found that contacts felt the fear of being stigmatised after being visited by a team of people in branded vehicles. This created embarrassment and fear of other community members finding out they had come in contact with a COVID-19 positive. In other parts of Ghana, contacts have recounted similar abused experiences by community members and others to not buy or sell to them.^23^ This could be one of the reasons for the bad treatment contacts gave to contact tracers who tried to visit them in their homes to follow up on them.

To overcome the impact of stigma, there is the need to engage community members in demystifying stigma.^24^ During our engagement with key stakeholders, we were able to get their buy-in and educate them on reducing stigma in the community. Again, community leaders have been found to play a major role in preventing stigma.^21^ This is because community members turn to respect them and trust the information they provide. Getting the buy-in of their stakeholders would mean they are serving as agents of change in the community. The stigma of COVID-19 has generally been found to be propagated by fear of the unknown nature of the virus.^25^

Most community members have limited information on COVID-19 and therefore end up stigmatising others out of ignorance. The intervention of community education and sensitisation was carried out to allay the fears of community members to helped them understand what COVID-19 is and the fact that everyone remains at risk of contracting it. Through the education, community members had their questions addressed.

Others opted for voluntary screening after understanding what COVID-19 was. Educating and sensitising community members can build a strong social support structure for recovered cases and their family members. This would help reduce the psychological effects and mental health issues attached to the stigma.^23^

Health workers have been found to play a part in reducing COVID-19 stigma. Based on experiences shared, the district modified its approach to contact tracing and other COVID-19 activities.

The impact of stigma on the individual and the society can be approached through multifaceted strategies like effective communication, stakeholder and community engagement, education, counselling and psychological support. From the implementation of the interventions, the issue of stigma was reduced in the community.

## Conclusion

In response to challenges faced by contact tracers and case managers, we observed deeply entrenched stigma to COVID -19 positive cases and their contacts in the community. Chiefly they avoided them, used derogatory words and insults. We found cases and contacts were stigmatised by family, friends, community and work colleagues. The presence of security personnel as a member of outbreak investigation teams and COVID-19 branded vehicles also fueled the stigmatisation.

## Figures and Tables

**Table 1:** Major themes from interviews with cases and contact

Constituents	Main themes identified	Frequency N(%)
**Cases**		
	Fear	13(65.0)
	Rejection	3(15.0)
	Shame	1(5.0)
	frustration	3(15.0)
**Contacts**		
	Fear	7(70.0)
	insecurity	3(30.0)
